# Supporting resident well-being on and outside the ICU during the COVID-19 pandemic: the use and value of institutional interventions and individual strategies

**DOI:** 10.1080/10872981.2021.1978129

**Published:** 2021-09-21

**Authors:** Renée A. Scheepers, Suzanne E. Geerlings, Mirja van der Meulen, Kiki Lombarts

**Affiliations:** a Erasmus School of Health Policy and Management, Erasmus University of Rotterdam, Rotterdam, The Netherlands; b Department of Internal Medicine, Amsterdam University Medical Center, Amsterdam, The Netherlands; c Professional Performance & Compassionate Care Research Group, Department of Medical Psychology, Amsterdam University Medical Center, University of Amsterdam, Amsterdam, The Netherlands; d Program for Medical Education Innovation and Research (Premier), Department of Medicine, Nyu School of Medicine, New York, New York, USA; e 2020–21 Presence-CASBS Fellow at the Center for Advanced Study of the Behavioral Sciences, Stanford University, California, USA

**Keywords:** Well-being, residents, medical education, Covid-19 pandemic, interventions, organizational support

## Abstract

During the COVID-19 pandemic, resident well-being has been shown to be at risk, which may interfere with residents’ process of professional development during their educational trajectory. Therefore, we developed a well-being program for residents, aimed to help residents maintain their well-being during the COVID-19 pandemic. We explored residents’ perceptions of their well-being as well as their perceived support of the well-being program during the COVID-19 pandemic. We invited all internal medicine residents and residents working in the ICU (N = 203) of one academic medical center to participate. The well-being program included a combination of (1) well-being measurements and (2) organizational support. The repeated well-being measurements involved a well-being survey on six measurement points from April to June 2020, and organizational support combined the provision of institutional interventions and promotion of individual strategies to help residents maintain their well-being during a pandemic. In total, 103 residents (50.1%) participated, showing that residents working in the ICU reported significantly lower levels of mental well-being than residents not working on the ICU. Furthermore, residents did not perceive the institutional interventions to benefit their well-being, while residents’ reported engagement in individual strategies was significantly positively associated with their well-being. As ICU residents reported lower levels of mental well-being, well-being programs need to address ICU-specific stressors while enhancing supervision and peer support. Furthermore, the individual strategies of the well-being program should be tailored to residents’ well-being needs as these were positively associated with resident well-being.

## Background

The well-being of residents has been persistently at risk since the onset of the COVID-19 pandemic [1,2]. Excessive workloads, a lack of time to rest or fear of infecting family members during the COVID-19 pandemic could all threaten residents’ well-being. Residents working at the ICU faced greater fears of infection, communication challenges, complete isolation and unusual additional tasks. This is especially alarming as poor well-being may hinder residents’ ability to deliver optimal patient care and may interfere with residents’ process of professional development during their educational trajectory[3]. According to the Accreditation Council for Graduate Medical Education (ACGME), resident well-being should be supported during their education[4], which is especially vital when well-being is at risk during (recovery phase of) the COVID-19 pandemic[5].

During previous pandemics (e.g., Ebola), resident well-being has been supported by interventions addressing self-care (e.g., exercise), reflective counselling or stress management[6]. During the COVID-19 pandemic, health-care professionals’ well-being has been supported by organizational interventions (e.g., telephone-based or peer support, or mental health specialist outreach) or by promoting individual strategies (e.g., seeking professional help) [7,8]. However, it is unknown how organizational interventions and individual strategies can be integrated into well-being programs for residents, and how residents perceive the support of these programs in the process of maintaining their well-being during the pandemic. Therefore, we included organizational interventions and individual strategies in a well-being program for residents during the COVID-19 pandemic.

## Objective

We explored residents’ perceptions of their well-being and their perceived support of the well-being program during the COVID-19 pandemic. The findings may be instrumental for hospitals preparing or fine-tuning well-being programs for residents during and beyond times of crisis.

## Methods

### Setting and participants

This study was conducted at one academic medical center in the Netherlands, during the first wave of COVID-19 from mid-March 2020 to June 2020. All internal medicine residents and residents working on the ICU (*N* = 203) were invited to participate in the well-being program, of which 103 (51%) residents positively responded. Ethical approval was waived by the Medical Ethics Committee of the Amsterdam Academic Medical Center.

### Interventions

The well-being program aimed to help residents maintain their well-being during the COVID-19 pandemic through a (1) repeated well-being measurement and (2) organizational support of resident well-being.

1. Repeated well-being measurement

All residents were invited to complete a well-being survey on six moments, chosen to allow for an immediate response in case of well-being decrease: April 6, 10, 17, 24, May 8 and 5 June 2020. The survey included questions about residents’ work location (ICU, COVID- or non-COVID unit) and well-being measures that were selected to fit a two-minute survey – acknowledging time pressures and long working hours – using single-item measures on physical, mental and emotional well-being, rated on a 5-point Likert scale (see supplementary file). Furthermore, the survey included additional questions inquiring on residents’ participation in organizational support of well-being and its perceived usefulness.

1. Organizational support of resident well-being

Organizational support for resident well-being consisted of two elements. First, providing residents with possibilities to engage in institutional interventions, including existing peer group meetings and newly developed 24/7 hospital-wide peer support, daily guided debriefings under supervision of a medical psychologist after the patient handover, two lectures on crisis management by a medical psychologist and trauma expert, information flyers, and a 24/7 telephone line for psychological support (see Box 1). These were presented at the homepage of the medical center’s intranet, during meetings of the Hospital-wide Residency Training Committee and the internal medicine or ICU department. Second, informing residents, through lectures and flyers, about individual strategies to help maintain well-being during a pandemic. The strategies suggested ranged from engaging in sporting activities, to walking, meditation, engaging in (online) social activities or seeking professional help.

**Box 1.** Institutional interventions involved in the well-being program

Institutional interventions	Approach	Self-reported use, in total (N, %) from 103 residents	Self-reported use of 45 ICU residents (N, %)	Self-reported use of 58 non-ICU residents (N, %)
Peer group meetings for residents	Group sessions with limited members, available both online and face-to-face	28 (28%)	9 (20%)	19 (33%)
Hospital-wide peer support	Support by trained and experienced medical specialists to ‘check in’ and collegially sit together with a resident; directly available (within 24 hours) for up to maximum three sessions; no therapeutic goals.	2 (2%)	2 (4%)	0 (0%)
Lectures on crisis management by a medical psychologist and trauma expert	Real-life and online lectures by a (war and pandemic) trauma expert sharing knowledge about how health-care professionals tend to respond to trauma and offering guidance in how to deal with the COVID-19 crisis as a health-care professional.	44 (43%)	18 (40%)	26 (45%)
Well-being Crisis Flyer	Provision of a flyer offering information and tips and tricks on how to stay well in times of crisis	29 (28%)	12 (27%)	17 (29%)
Guided debriefing by medical psychologist during the patient handover	Guided debriefing sessions with the whole health-care team after night shifts; encouraging to share experiences to emotionally and mentally distress.	17 (17%)	5 (11%)	12 (21%)
24/7 telephone line for psychological support	Telephone support was available for direct psychological help.	0 (0%)	0 (0%)	0 (0%)

### Analyses

We performed multilevel regression analyses – within the RStudio environment 1.2.5042 using ‘psych’ (version 1.9.12) and ‘lme4’ (version 1.1–23) packages – to account for repeated measures nested within residents. We fitted models per dependent variable (mental, physical and emotional well-being) to determine associations between well-being and organizational support, i.e., the use of specific institutional interventions or individual strategies, and to determine well-being differences between residents working at the ICU and residents working in other services.

## Results

In total, 254 surveys covering the six moments were completed; response rates varied from 46.8% at the start to 19.7% at the end of the first wave (Table 1).

1. Repeated well-being measurement

**Table 1.** Response rates of participants throughout the study time (from April 6 to 5 June 2020)

	Date	Complete	Incomplete	No response	Residents invited	Response rate	N of participating residents working on ICU
1	6 April	44	2	48	94	46,8%	9 (20%)
2	10 April	32	3	81	116	27,6%	8 (25%)
3	17 April	44	3	98	145	30,3%	15 (34%)
4	24 April	46	2	155	203	22,7%	21 (46%)
5	8 May	48	2	153	203	23,6%	13 (27%)
6	5 June	40	1	162	203	19,7%	9 (23%)
Total surveys		254	13	697	964	**26,3%**	**75 (30%)**
Total N of residents		103	N/A	100	203	**50,7%**	45 (44%, with 16 residents switching from ICU to non ICU)

**Figure 1.**
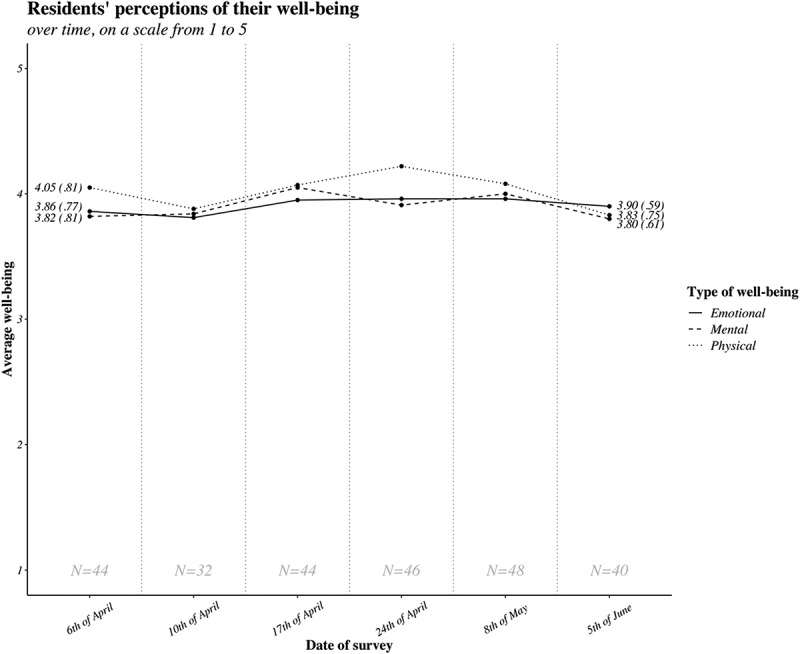
Residents’ average perception of their mental, physical and emotional well-being throughout the study period (April–June), on a scale from 1 to 5

Resident well-being did not significantly change over the study period; all regression coefficients of the different time periods were non-significant for all types of residents’ well-being (Figure 1, supplementary files). Residents working on the ICU reported lower levels of mental well-being: 3.75 (SD = .96) compared to 3.98 (SD = .76) of residents not working on the ICU (*b* = .26, t(142) = 2.07, p < .04). No differences were found between residents working in the ICU compared to those not working in the ICU on physical or emotional well-being.

1. Organizational support of resident well-being

Values represent the mean (SD) of residents’ well-being at the first measurement, and last measurement.

No significant associations between institutional interventions and residents’ perceived (physical, mental or emotional) well-being were found. Residents’ reported engagement in individual strategies was significantly positively associated with their mental well-being, physical well-being and emotional well-being. The strategy ‘seeking professional help’ was associated with less emotional well-being (b = −.67, t(185) = −3.41, p < .001), and ‘taking a walk’ and ‘exercise/physical activity’ were associated with more emotional well-being (b = .28 t(204) = 2.40, p = .02, and b = .35 t(199) = 2.56, p = .01, respectively). Furthermore, ‘taking a walk’ was associated with more mental well-being (b = .32, t(202) = 2.23, p = .02), and ‘exercise/physical activity’ was associated with more physical well-being (b = .41, t(208) = 3.70, p < .001).

## Discussion

Despite the pressures of COVID-19 on residents’ learning trajectories and work in daily practice, residents report rather stable well-being levels. Residents working at the ICU reported lower levels of well-being than residents working outside the ICU. Residents employing individual strategies focused on a healthy lifestyle reported higher levels of physical, mental and emotional well-being. Individual activities, mostly taking place outside the work environment and being under direct control of residents, may explain this finding. We recommend health-care organizations to further look into the potential benefits of cultivating individual strategies in residents’ leisure time and during work hours, i.e., walking lunches or exercise activities. Incorporating these activities into daily practice may be challenging due to excessive workloads and should be tailored to local circumstances during a (post-) pandemic.

The COVID-19 pandemic may place continued strain on resident well-being, now and during post-pandemic upscaling of regular care. Since health-care organizations have the responsibility to maximize resident well-being, they should provide them with sufficient recovery possibilities, information on well-being supporting individual strategies, and ideally combine these with organizational support [9].

## Supplementary Material

Supplemental Material
