# Supplementary material for: Supporting resident well-being on and outside the ICU during the COVID-19 pandemic: the use and value of institutional interventions and individual strategies
Source: Med Educ Online. 2021 Sep 21;26(1):1978129. doi: 10.1080/10872981.2021.1978129 (PMC8462914; doi:10.1080/10872981.2021.1978129)

Figure 1. Residents’ average perception of their mental, physical and emotional well-being throughout the study period (April – June), on a scale from 1 to 5.
*Values represent the mean (SD) of residents’ well-being at the first measurement, and last measurement.*


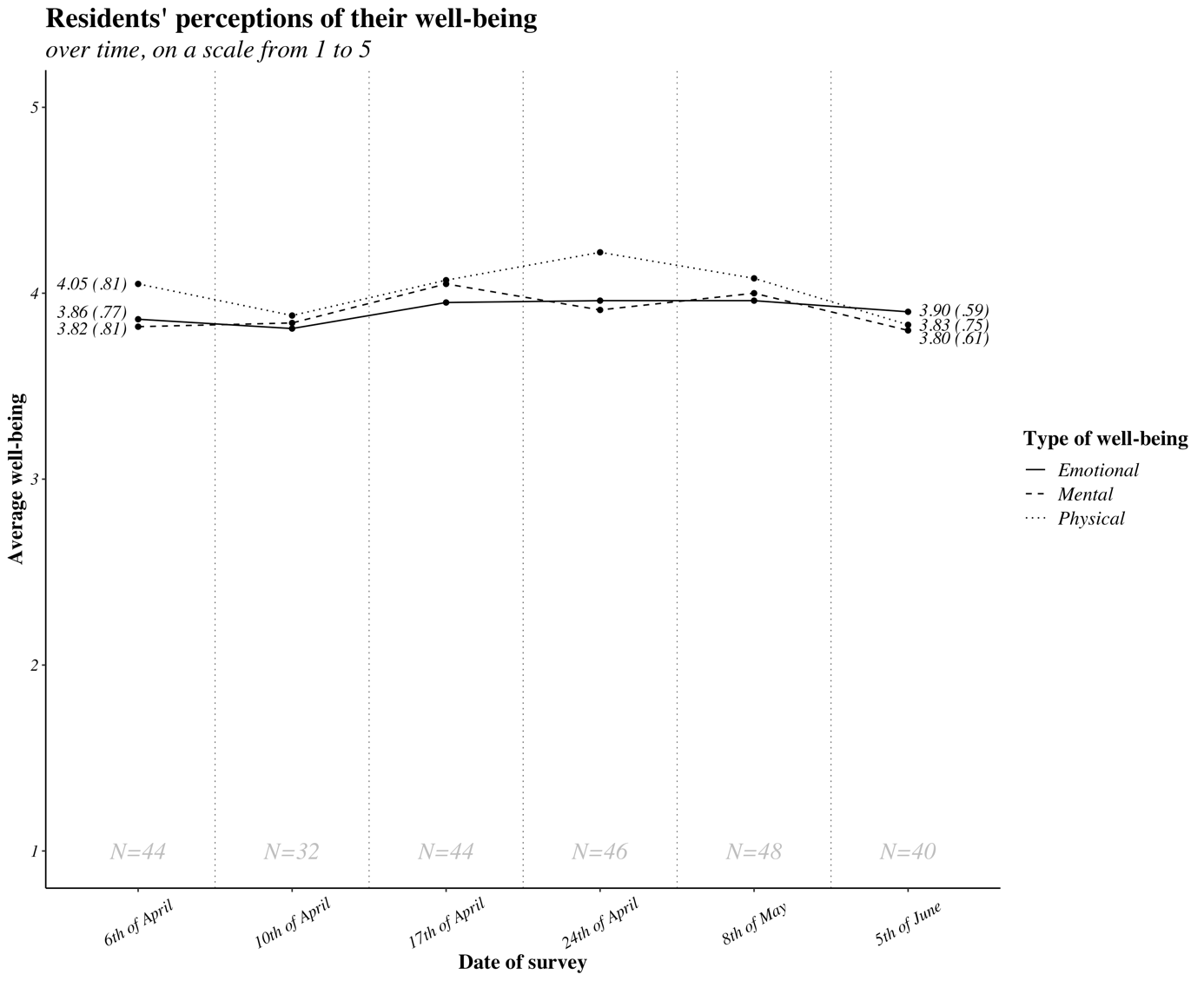

Supplement: Supplemental Material [file ZMEO_A_1978129_SM2659.zip › Supplementray/Figure 1 Supplementary file.docx]
