# Supplementary material for: Supporting resident well-being on and outside the ICU during the COVID-19 pandemic: the use and value of institutional interventions and individual strategies
Source: Med Educ Online. 2021 Sep 21;26(1):1978129. doi: 10.1080/10872981.2021.1978129 (PMC8462914; doi:10.1080/10872981.2021.1978129)
Supplement: Supplemental Material [file ZMEO_A_1978129_SM2659.zip › Supplementray/Supplementary file_Survey_MEOnline .docx]

**Supplementary files**

**Supplementary file 1. Well-being survey for residents**

In which specialty are you trained?

- Internal medicine
- Cardiology
- Pulmonology
- Other

Did you work on the ICU last week?

- Yes
- No

Sex

- Male
- Female

How fit and healthy are you at this moment?

- 1 - Totally not fit (red smiley)
- 2
- 3
- 4
- 5 – Super fit (green smiley)

How physically healthy are you at this moment?

- 1 - Totally not fit (red smiley)
- 2
- 3
- 4
- 5 – Super fit (green smiley)

How mentally healthy are you at this moment?

- 1 - Totally not fit (red smiley)
- 2
- 3
- 4
- 5 – Super fit (green smiley)

How emotionally healthy are you at this moment?

- 1 - Totally not fit (red smiley)
- 2
- 3
- 4
- 5 – Super fit (green smiley)

The institution provides different intervention to support your well-being. In which of these interventions did you participate last week?

- Guided peer group meetings for residents
- Telephone-based support
- Peer support of the institutional Peer support team
- Tips of the Ministry of Defense (flyer)
- Guided debriefing by a medical psychologist during the patient handover
- Webinar about COVID-19
- Other institutional interventions, i.e. [open text box]

Which activities have you employed last week to maintain your well-being in these times?

- Sporting activities at home / outdoors
- Taking a walk
- Yoga at home
- Meditation
- Online social activities with friends / family
- Seeking professional help outside the institution
- Other activities, i.e. [open text box]

Do you have tips for your colleagues to stay physically, mentally and emotionally healthy?

- Open text box

Many thanks for your participation.

[End of survey]
